# Money makes you reveal more: consequences of monetary cues on preferential disclosure of personal information

**DOI:** 10.3389/fpsyg.2013.00839

**Published:** 2013-11-11

**Authors:** Sumitava Mukherjee, Jaison A. Manjaly, Maithilee Nargundkar

**Affiliations:** Cognitive Science Program, Indian Institute of Technology GandhinagarAhmedabad, India

**Keywords:** self-disclosure, privacy, money, priming, preference

## Abstract

With continuous growth in information aggregation and dissemination, studies on privacy preferences are important to understand what makes people reveal information about them. Previous studies have demonstrated that short-term gains and possible monetary rewards make people risk disclosing information. Given the malleability of privacy preferences and the ubiquitous monetary cues in daily lives, we measured the contextual effect of reminding people about money on their privacy disclosure preferences. In experiment 1, we found that priming money increased willingness to disclose their personal information that could be shared with an online shopping website. Beyond stated willingness, experiment 2 tested whether priming money increases propensity for actually giving out personal information. Across both experiments, we found that priming money increases both the reported willingness and the actual disclosure of personal information. Our results imply that not only do short-term rewards make people trade-off personal security and privacy, but also mere exposure to money increases self-disclosure.

## Introduction

We increasingly interact and exchange important information with strangers as part of various daily transactions. Although self-disclosure enhances personal relationships, including trust, it is also inherently risky as making personal information known to others trades off personal privacy (Alter and Oppenheimer, 2009). The virtual social space (such as Facebook™, Google Hangout™) has been amassing personal information from its users, part of which can be accessed by strangers. Although the social networking companies update their users on privacy policies, users seldom pay attention to the warnings. Interestingly, most people claim to be concerned about privacy, but often give out information (the paradox of privacy; Norberg et al., 2004; Norberg and Horne, 2007). The inadvertent disclosure of information by the customers could end up with online predators and phishing agents. At times, this can involve substantive costs to both the customers and companies (Huffington Post, 2012).

Self-disclosure poses many threats that can be both financial and social, including frauds, thefts, and complicated doctor-patient relations (Epstein et al., 1998; Alter and Oppenheimer, 2009). Thus, it is important to understand what causes people to reveal their personal information, especially the incidental effects of common environmental cues. Although much of social science and many privacy models (e.g., Dinev and Hart, 2006) assume privacy preferences are stable over time, information disclosure preferences have been shown to vary situationally and across time (Acquisti, 2009). Malleable privacy preferences can be contextually affected by cues in the environment. Some theories (such as the Contextual Integrity Theory of privacy; Nissenbaum, 2004) factor the role of context in privacy, but they treat context to mean differing norms and social roles that mediate information disclosure behavior. Studies have also highlighted the contextual effects of framing (John et al., 2011) and the order of presenting questions (Acquisti et al., 2012). In general, different contextual variables seem to affect information disclosure.

We observed that there is surprisingly little research on the effect of task-unrelated situational cues. For example, online shopping sites embedded with monetary cues (like discounts) have been aggressively collecting personal data from users. Even social networking sites or chat rooms, where personal information is solicited, often display advertisements with monetary cues. Priming a concept or emotion can significantly affect preferences, judgments, and decisions (Tversky and Simonson, 1993). In this report, we sought to mitigate the scarcity of literature on the effect of situational primes and focused on how monetary primes influence privacy disclosure preferences.

Contrary to the rational assumption, consumers often trade off privacy for short-term benefits (Acquisti and Grossklags, 2005). What then would be the effect of simply priming the idea of money? Even in the absence of actual rewards, would cues of money affect self-disclosure preferences such that it trades off privacy?

Among the few published studies related to economic considerations in privacy preferences, Grossklags and Acquisti (2007) have shown that people are less willing to pay for protecting their data compared to their willingness to accept money in exchange for their personal information. Considering the psychological aspect of money, implicit reminders of money affect various types of personal and interpersonal processes (Vohs et al., 2008) as money induces a feeling of self-sufficiency (Vohs et al., 2006, 2008) due to which people are more likely to opt for isolated goal pursuits. Money has also been psychologically linked to strength (Vohs et al., 2008; Zhou et al., 2009), which sometimes results in preferences for non-interference (Liu et al., 2011). We are not aware of any study that measured the effect of priming money on disclosure of personal information. Monetary cues are commonly present during transactions of information in both online and during direct social interactions. For example, paying for using Internet in kiosks, making online payment of bills or viewing billboard advertisements with lucrative offers remind people about money, while they might be talking about themselves.

Psychologically, reminders of money induce a self-sufficiency bias (Vohs et al., 2006). In such situations, people might tend to underestimate the potential harm information disclosure could cause, and hence, become more inclined toward disclosing personal information. Moreover, immediate gratification obtained from money (Grossklags and Acquisti, 2007) or desirable objects (Yap et al., 2009) influences one to disclose information. We intended to examine whether mere reminders of money (without any actual reward) could increase willingness to disclose individual information to strangers.

## Overview of the studies

In the first experiment, we tested whether monetary cues increase willingness to disclose information. The second experiment measured actual disclosure behavior among the money-primed and control groups. The overall hypothesis was that money would increase self-disclosure of personal information.

In order to arrive at a pool of personal information items for the present population, we conducted a pretest by asking a group of 20 naïve adults to list which information they treated as private or personal. Later a separate group of 89 students from the same population rated the extent to which they would be willing to disclose that information to a stranger, if asked for. The items used in the following studies belonged to this list of personal information. In all the experiments, we probed a group of participants for suspicion and checked whether they understood the task. All participants were debriefed briefly after the experiment.

## Experiment 1

Participants were requested to complete a paper based “consumer survey” that asked them for their willingness to disclose a number of personal information items varying in severity of intrusiveness. We manipulated the prime using the background such that one group saw a picture of money while another group saw a scrambled version of the same picture.

### Participants

Eighty-three undergraduate students (females = 23; mean age = 21 years) from Indian Institute of Technology Gandhinagar (where medium of instruction is English) were requested to participate in a “consumer survey.” After an informed consent, an almost equal number of subjects were randomly assigned to groups by an experimenter blind to the conditions.

### Procedure

Participants were told that we were aggregating some information from different people to share it with an online shopping site. On a paper based form, they were required to rate their willingness to disclose those individual items on a Likert scale of 1 (*not at all willing*) to 7 (*absolutely willing*). They were explicitly told that this information might be shared with people whom they do not know. The items were gender, skin complexion, height, weight, waist size, date of birth, name, roll number in class, mobile number, and email. Following previous researchers (Vohs et al., 2008; Quoidbach et al., 2010), the background picture of the form manipulated the prime. The experimental group (*n* = 42) saw a picture of Indian currency notes printed in color in the background, while the control group (*n* = 41) saw a scrambled version of the same picture in the background (see Figures S1, S2 in supplementary material).

### Results

We analyzed the data after removing three participants (one was an outlier and two of them did not fill all the items in the form). The privacy-index (Cronbach's alpha = 0.76) was created by aggregating the ratings across all the items (for means of individual items, see Table S1 in supplementary material). The One-Way ANOVA showed a main effect of the prime, *F*_(1, 78)_ = 4.85, *p* = 0.03, η^2^ = 0.05^1^, where the willingness to disclose information was higher in the money group compared to the control group (see Table S3 in supplementary material). This shows that in the presence of money people are more willing to disclose personal information.

## Experiment 2

Beyond hypothetical willingness to disclose, this experiment was designed to measure how monetary cues could affect actual disclosure of personal information^2^. We had previously suggested that one possible mechanism through which money might prime more disclosure is by inducing a sense of self-sufficiency (Vohs et al., 2008), and hence, we reasoned that it should enhance feelings of self-efficacy. The commonly used generic self-efficacy scale^3^ (Schwarzer and Jerusalem, 1995) was used to measure self-efficacy. It was hypothesized that those who were primed with money will feel that they are sufficiently capable to deal with life situations, and hence, will report higher levels of self-efficacy.

### Participants

Eighty-eight undergraduate students (females = 24; mean age = 20 years) from Indian Institute of Technology Gandhinagar voluntarily participated in the study in response to a request.

### Procedure

Participants were told that they had to complete two small, unrelated tasks. The first study was framed as a preliminary understanding of people's behavior on the Internet (which was our dependent variable for measuring disclosure behavior). They were told that we were aggregating some information from different people to share it with an online shopping website. We clearly informed them that this information might be shared with people whom they did not know and all the items are optional. Participants were asked to enter the following information about them on a form: gender, skin complexion, height, weight, waist size, date of birth, name, roll number in class, mobile number, and email address (but answering the items were optional).

For one group of participants (*n* = 44), the form had a picture of money in the background (money group), while for the other group (*n* = 44), there was a scrambled version of the same money picture in the background (control group). The form was similar to experiment 1 (see supplementary material), but the only difference being that in place of willingness for disclosure, participants were actually asked to fill up the information in corresponding text boxes.

After they filled up the form, we asked them to complete another small, unrelated task that was framed as a study on personality. Here, we presented them with the 10 items of the general self-efficacy scale (Schwarzer and Jerusalem, 1995; Luszczynska et al., 2005) on a normal paper without background images that measured optimistic self-beliefs to cope with different situations in life.

### Results

Their responses to the disclosure of personal information was reverse coded on a 1–0 metric, where subjects received a score of 0, when they did not reveal that piece of information, and received a 1, when they did reveal. For example, if they filled in their email address, it was counted as 1, and if they left it blank, it was coded as 0. Thus, those who revealed more number of items had a higher score (see Table S2 in supplementary information for details of descriptive statistics). The univariate ANOVA revealed a main effect of the prime, *F*_(1, 86)_ = 4.66, *p* = 0.03, η^2^ = 0.05 with the money group resulting in a higher score compared to controls, showing that priming money made participants opt for actually revealing more information^4^ (see Table S3 in supplementary material).

For self-efficacy, the items were summed to an aggregate score (Schwarzer and Jerusalem, 1995). It was found that the money primed group reported higher feelings of self-efficacy compared to the control group, *F*_(1, 86)_ = 4.86, *p* = 0.03, η^2^ = 0.05 (see Table S3). This provides some preliminary evidence that money increases the sense of self-efficacy. We believe that such a feeling potentially enhances belief in the self as being capable to deal with any situation in life, in-turn increasing self-disclosure, even though doing so could be risky.

## General discussion

We intended to study how simple monetary cues affect self-disclosure preferences. Both experiments showed that reminders of money increase propensity of self-disclosure. Money might induce abstract forms of gratification and control, thereby influencing preferential disclosure of personal information. Further, promotion or approach oriented cues like money, elicit a risky explorative style (Friedman and Förster, 2001), and hence, monetary cues could induce more risky behavior of disclosing personal information. One mechanism through which money increases self-disclosure could be through a heightened sense of self-efficacy to deal with (risky) future scenarios. Our results are the first to show that simple commonplace cues of money can influence self-disclosure preferences.

Social relationships and access to social resources are heavily dependent on sharing of information, which could be partly habitual. Inhibiting this habitual action of self-disclosure will require exercise of self-control, which is beneficial if long-term risk of misuse could be mitigated. We believe money makes people focus on immediate short-term gains, which is common for motivational stimuli (Kim and Zauberman, 2012; e.g., Van den Bergh et al., 2008), thus undermining long-term risks of misusing their information. This myopic focus induced by motivational stimuli (Van den Bergh et al., 2008) such as money, results in lesser self-control and hence, increased disclosure. Further, the self-sufficiency bias induced by money (Vohs et al., 2006, 2008) possibly underestimates possible risks of information disclosure by making people less vigilant, and thereby, increasing informational disclosure. We obtained some tentative evidence that priming money makes people believe that they can deal with a variety of situations in life (increase in self-efficacy). Such a feeling can influence disclosing about oneself either due to underestimation of potentials future costs or to project more about oneself.

Most current privacy models have so far considered variables such as risk, trust, perception of control, relevance of information, perceived usefulness of the product/services, and monetary rewards (e.g., the privacy calculus model; Dinev and Hart, 2006). Results from these experiments highlight the role of contextual variables as powerful sources (also see John et al., 2011). Thus, privacy preferences are not a mere outcome of the risk-benefit analysis, but are contextually moderated by perceptual information totally unrelated to the task. In that sense, preferences are embodied in the current environment. Existing models of privacy may require modification to include such situational context effects.

There are some limitations in the current study that should be taken into account while interpreting the results. Personal information is categorical to some extent and is moderated by individual preferences. So, our results should not be interpreted to mean monetary cues would affect all kinds of informational disclosure. In fact, on closer examination of the data (Tables S1, S2), it can be seen that for more personal (such as mobile phone number) or comparatively less personal information (such as gender), there is no effect of monetary primes on both willingness to disclose and actual disclosure. Further, the order of questions (Acquisti et al., 2012) can also moderate the effects. Our studies used a small sample of the population (youths in the age range of 18–22 with a slight larger number of males from India), and hence, it is not clear whether these effects would hold for populations of a different age and country. There were also no explicit manipulation checks of the priming method. Part of the difficulty to do so lies in there not being a clear way to check for priming manipulations of the sort we used, and that is perhaps why previous scholarship on monetary primes (e.g., Vohs et al., 2006) have also not included such manipulation checks. As the prime was salient and clearly visible, it should be relatively clear that the concept of money was indeed activated in their minds. A related point is about the control image. Apart from the experimental and control groups differing in exposure to the concept of money, there is another difference. The experimental group saw a meaningful stimuli (the money image) while the control group saw an ecologically non-meaningful image (scrambled version of money image). Thus, it is possible that this difference could be contributing in part to the results. Note, however, that this difference does not invalidate the difference between money and non-money cues. Finally, self-sufficiency and a risky promotion orientation are probable explanations, but are not exhaustive. One important complementary explanation could be that money emphasizes input and output through equity (Vohs et al., 2008). It is hence, possible that priming money makes people disclose more with an implicit assumption that doing so would encourage more exchanges between themselves and another party (stranger). This means giving out information about the self would lead them to receiving something in return (the market exchange mode of thought). The literature on the influences of money is just staring to grow, and we need more studies that could shed light on the mechanistic explanations of the psychological consequences of priming money.

In sum, we find evidence that monetary cues increase propensity to reveal personal information. Such a finding has many implications, as self-disclosure and money are part of our daily functioning. People are frequently reminded of prices, discounts, and offers among other monetary cues due to aggressive advertisement campaigns of products and services. As information disclosure may frequently occur in the presence of such primes, policies (Sunstein and Thaler, 2003) could factor such possibilities. Beyond social preferences and self-disclosure, the centrality of monetary cues in daily life implies that we need to understand how money influences our social and cognitive processing in more detail.

## Author contributions

Sumitava Mukherjee planned the research, Sumitava Mukherjee and Jaison A. Manjaly designed the studies, Maithilee Nargundkar and Sumitava Mukherjee analyzed the data, Sumitava Mukherjee and Maithilee Nargundkar wrote the manuscript along with inputs from Jaison A. Manjaly during revisions.

### Conflict of interest statement

The authors declare that the research was conducted in the absence of any commercial or financial relationships that could be construed as a potential conflict of interest.

## Footnotes

^1^The Levene's test for homogeneity of variances was not significant, thus, showing that our groups were homogeneous, *F*_(1, 78)_ = 0.004, *p* = 0.947.

^2^We thank the reviewers and the editors for suggesting this study.

^3^The scale is now available in multiple languages at http://userpage.fu-berlin.de/health/selfscal.htm

^4^The Levene's test of equality of variances showed that our groups were not significantly different and were homogeneous, *F*_(1, 86)_ = 2.10, *p* = 0.15.

## Supplementary material

Figure S1 **Form for money group in experiment 1**.

Figure S2 **Form for control group in experiment 1**.

Table S1 **Mean willingness to disclose in Experiment 1**.

Table S2 **Number of participants who disclosed the following information in experiment 2**.

Table S3 **Results of the One-Way ANOVA for experiment 1 (mean privacy-disclosure index) and Experiment 2 (actual disclosure and self-efficacy)**.
