# Supplementary material for: Money makes you reveal more: consequences of monetary cues on preferential disclosure of personal information
Source: Front Psychol. 2013 Nov 11;4:839. doi: 10.3389/fpsyg.2013.00839 (PMC3822294; doi:10.3389/fpsyg.2013.00839)
Supplement: Figure S1 — Form for money group in experiment 1. [file DataSheet1.PDF]

Supplementary information

Table S1. Mean willingness to disclose in Experiment 1.

**Information item**	**Conditions**	
	Money	Control
Gender	6.65 (0.9)	6.35 (1.2)
Skin complexion	5.68 (1.7)	4.85 (2.2)
Height	6.18 (1.5)	5.40 (1.7)
Weight	5.78 (1.7)	4.80 (1.9)
Waist size	4.52 (1.9)	4.19 (2.0)
Date of birth	4.68 (2.3)	4.16 (2.2)
Name	4.65 (2.3)	3.76 (2.1)
Roll number	3.31 (2.2)	3.30 (2.2)
Mobile number	2.84 (1.7)	2.16 (1.5)
Email	3.42 (1.9)	3.57 (2.1)
**Privacy Index**	**47.76 (1.2)**	**42.59 (1.1)**

Table S2. Number of participants who disclosed the following information in experiment 2.

**Information item**	**Number of participants who disclosed across conditions**	
	Money (total n = 44)	Control (total n = 44)
Gender	44	44
Height	41	42
Waist size	33	36
Name	38	30
Mobile number	28	16
Skin complexion	34	33
Weight	43	42
Date of birth	41	36
Roll number	38	25
Email	31	25

Table S3. Results of the one-way ANOVA for experiment 1 (mean privacy-disclosure index) and Experiment 2 (actual disclosure and self-efficacy)

	Mean (SD)				
	Money	Control	F	df	p
Privacy-disclosure index	47.76 (10.29)	42.59 (10.63)	4.85	78	.03*
Actual disclosure	8.43 (1.95)	7.47 (2.18)	4.66	86	.03*
Self-efficacy	2.83 (0.28)	2.64 (0.49)	4.86	86	.03*

*indicates $p < .05$

Instructions and Forms used in Experiment 1

Instruction:

We are aggregating some information from people to share it with an online shopping site. Please note that this information might be shared with people whom you do not know. Please rate your willingness to disclose the following information on a scale of 1 (not at all willing) to 7 (absolutely willing).

Figure 1. Form for money group in experiment 1

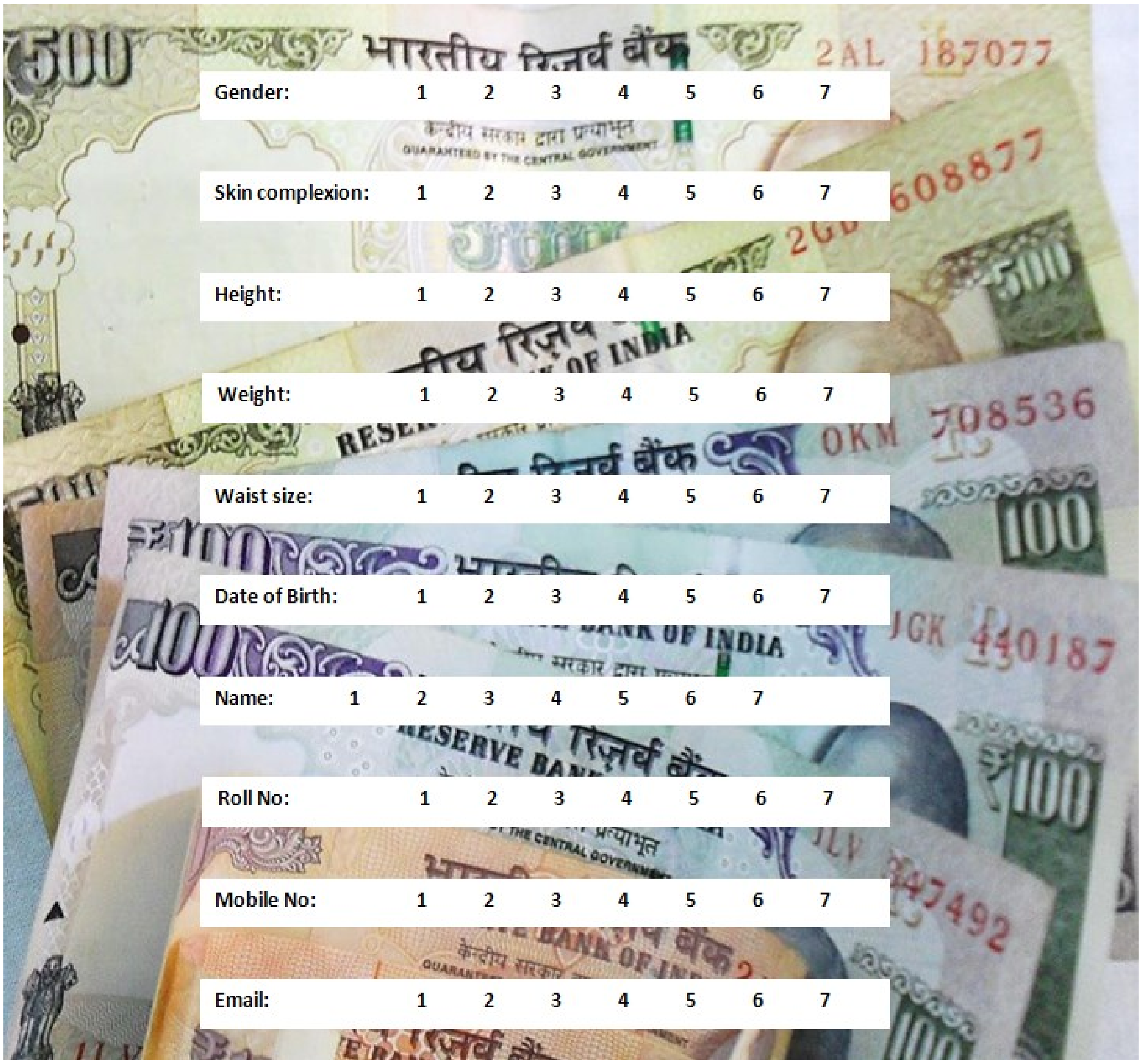

Gender:	1	2	3	4	5	6	7
Skin complexion:	1	2	3	4	5	6	7
Height:	1	2	3	4	5	6	7
Weight:	1	2	3	4	5	6	7
Waist size:	1	2	3	4	5	6	7
Date of Birth:	1	2	3	4	5	6	7
Name:	1	2	3	4	5	6	7
Roll No:	1	2	3	4	5	6	7
Mobile No:	1	2	3	4	5	6	7
Email:	1	2	3	4	5	6	7

Figure 2. Form for control group in experiment 1

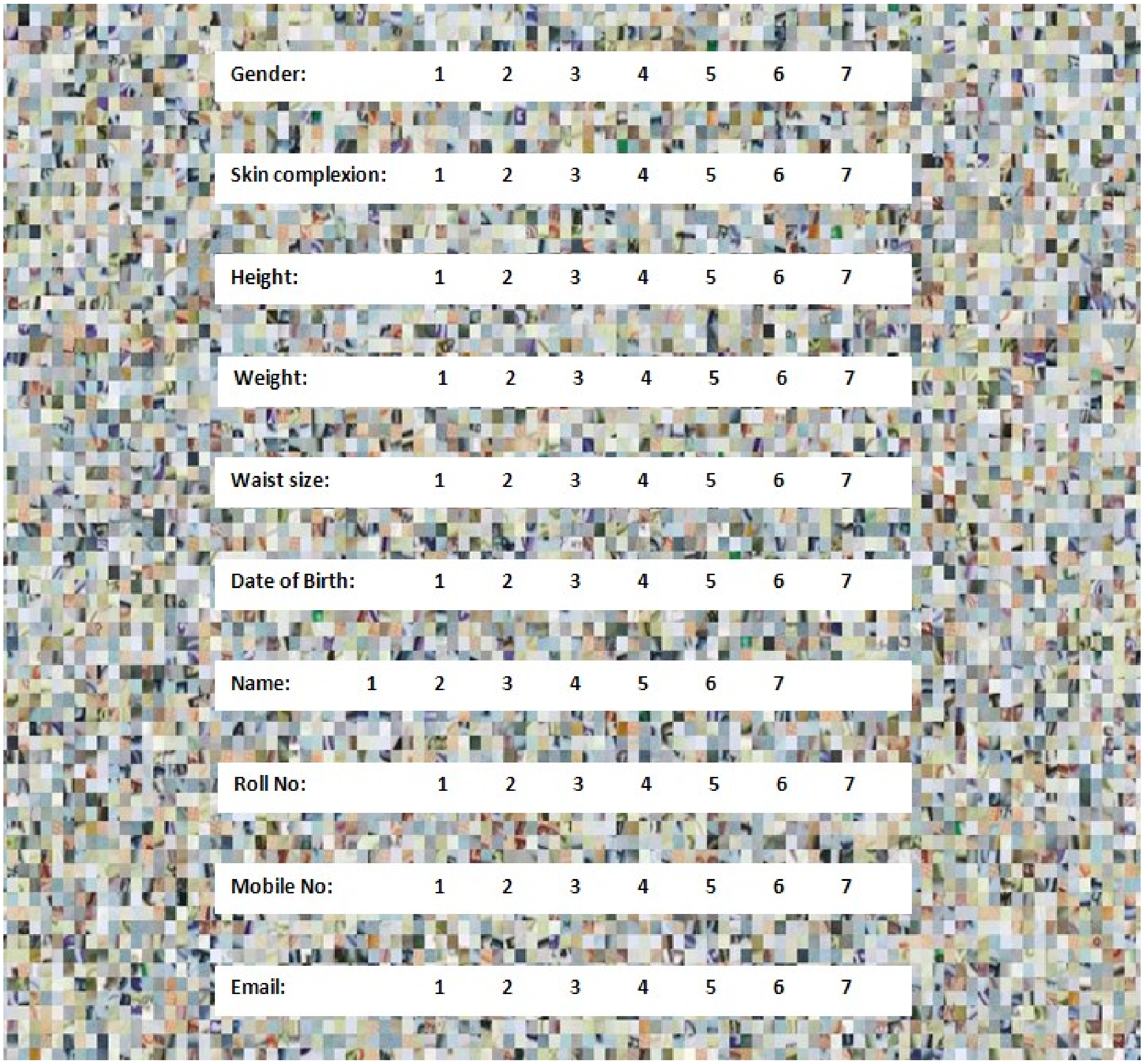

Gender:	1	2	3	4	5	6	7
Skin complexion:	1	2	3	4	5	6	7
Height:	1	2	3	4	5	6	7
Weight:	1	2	3	4	5	6	7
Waist size:	1	2	3	4	5	6	7
Date of Birth:	1	2	3	4	5	6	7
Name:	1	2	3	4	5	6	7
Roll No:	1	2	3	4	5	6	7
Mobile No:	1	2	3	4	5	6	7
Email:	1	2	3	4	5	6	7
